# Zika virus reservoirs: Implications for transmission, future outbreaks, drug and vaccine development

**DOI:** 10.12688/f1000research.12695.1

**Published:** 2017-10-17

**Authors:** Raj Kalkeri, Krishna K. Murthy

**Affiliations:** 1 Infectious Diseases Research, Southern Research, Frederick, MD, 21701, USA; 2 Independent Researcher, San Antonio, TX, 78249, USA

**Keywords:** Zika, Persistence, transmission, tissue reservoirs, drug and vaccine development

## Abstract

Zika virus (ZIKV) was recently declared as a ‘Global Health Emergency’ by the World Health Organization. Various tissue reservoirs of ZIKV in infected humans and animals models have been observed, the implications of which are not known. Compared to other Flaviviruses, sexual transmission and persistence in the genitourinary tract seem to be unique to ZIKV. ZIKV persistence and shedding in bodily secretions (e.g. saliva, semen) is a concern for potential disease spread and could pose challenges in diagnosis, regulatory guidelines and drug/vaccine development. Murine and non-human primate models could be useful to study the role of tissue reservoirs in the development of prophylactic or therapeutic strategies. There is a need for meta-analysis of the ZIKV infection and virus shedding data from infected patients and ZIKV animal models, and additional research is needed to fully comprehend the long term implications of tissue reservoirs on ZIKV disease pathogenesis and biology.

## Introduction

After the recent outbreak of Zika virus (ZIKV) infection in Brazil and other countries, multiple studies have been published regarding various tissue reservoirs, including the eyes, nervous system, genitourinary tracts and placenta ( Table 1). A recent report of ZIKV transmission in renal and liver transplant patients with abnormal graft functions ^1^ poses a new challenge in screening organs intended for transplants and monitoring transplanted patients. Considering such new concerns for ZIKV, we have reviewed the existing literature about ZIKV tissue reservoirs in this article and provide our analysis of the potential impact of ZIKV reservoirs on transmission and challenges in development of medical countermeasures against ZIKV.

**Table 1.** ZIKV reservoirs reported in humans.

Organ	Confirmed in animal models
Eyes ^16, 42^	25
Kidney ^11^	Due to detection in urine and translation from mice studies.
Nervous Tissue ^6^	24, 27
Testes ^13, 17^	Due to detection in semen and translation from mice studies ^2, 18, 23^.
Placenta ^3, 6^	22, 43

## ZIKV tissue reservoirs

The role of immune-privileged sites (e.g. eyes, testes, fetal brain, and placenta) in harboring ZIKV has been previously reported ^2^, contributing to the viral persistence in these tissues. Successful infection and amplification of ZIKV *in vitro* in primary cell cultures derived from the placenta ^3^, kidney ^4^, nervous tissue ^5^, and amplification found in the brain of severely damaged fetus ^6^ suggests the potential ability of these tissue reservoirs to support both infection and amplification of ZIKV. Furthermore, that the reservoir-derived virus is infectious has been confirmed by sexual- and or transplant-related transmission of infection from non-viremic partners or donors ^7^. These studies raise the possibility that individuals with ZIKV reservoirs could serve as a potential source of endemic and recurrent outbreaks of infection. In addition, the prevalence of such reservoirs in immune compromised patients ^8^ is a major concern as the disease could spread to other organs rapidly and run a more aggressive course, in the absence of immune defenses of the body.

## ZIKV in body secretions

Interestingly, besides tissue reservoirs, few reports have been successful in the detection of ZIKV in the bodily secretions of infected patients, such as saliva ^9, 10^ urine ^9, 11^, semen and vaginal secretions ^12– 14^, breast milk ^15^ and conjunctival fluid ^16^. Semen and vaginal secretions are already reported to be involved in sexual transmission of ZIKV ^7^ in humans. Despite a recent report about the persistence of ZIKV in the semen for more than 6 months ^17^, the duration of the persistence in other bodily secretions (urine, saliva etc) is unclear at this time. Careful evaluation of the duration of the ZIKV shedding and its role in non-sexual transmission needs to be conducted. 

## *In vivo* models for ZIKV tissue reservoirs

Animal models for ZIKV have further confirmed some of these tissue reservoirs ^18– 25^. Tang *et al.* demonstrated the persistence of ZIKV in the vaginal washes as late as 10 days post-infection in lethal and sub-lethal mouse models after intravaginal inoculation of ZIKV ^26^. Ifnar1(-/-) mice showed sustained high viral loads in the brain, spinal cord and testes, confirming these tissue reservoirs ^21^. AG129 mice showed severe pathologies in the muscle and brain ^27^, confirming the presence of these reservoirs in mouse models.

ZIKV was shown to persist in the cerebrospinal fluid (CSF) and lymph nodes (LN) of infected rhesus monkeys weeks after virus clearance from the peripheral blood, urine and mucosal secretions ^28^. Similar observation of longer persistence of ZIKV in the hemolymphatic tissue of non-pregnant rhesus macaques infected with 2015 Brazilian ZIKV isolate was observed by Coffey and colleagues ^29^. ZIKV was also observed in the lacrimal fluid and parotid glands ^30^ of rhesus macaques after subcutaneous infection. ZIKV RNA was detectable in saliva, semen, urine of both rhesus and cynomolgus macaques ^20^. ZIKV infection in the pregnant rhesus macaques showed longer duration of persistence (57 days) compared to normal macaques (21 days) ^31^, suggesting differential persistence depending upon the pregnancy status.

A recent study showed the importance of autophagy on the vertical transmission of ZIKV in pregnant mice ^32^. The authors demonstrated the activity of an autophagy inhibitor approved for use in pregnant women (hydroxychloroquine) in attenuating the placental and fetal ZIKV infection and ameliorated adverse placental and fetal outcomes. Thus animal models could prove useful to study the importance of tissue reservoirs in ZIKV biology and pathogenesis, as well as evaluation of experimental drugs and vaccines.

## Relationship to other Flaviviruses

A review of the literature suggested similar tissue reservoirs observed for other Flaviviruses ( Table 2). Tick-borne encephalitis, West Nile Virus and Japanese Encephalitis Virus have varying durations of persistence ^33^. However, unlike other Flaviviruses, sexual transmission is unique to ZIKV infections ^7^. This is not surprising due to ZIKV’s persistence in the genitourinary tract ^34^. The presence of ZIKV in semen is without apparent disease symptoms ^35^, and can be for a period of more than 6 months ^17^. This indicates a need for inclusion of ZIKV in the screening panel for semen donors in addition to the current viruses in the panel (i.e. hepatitis B, C and HIV, as per the guidelines of American Society for Reproductive Medicine and Food and Drug Administration).

**Table 2.** Tissue reservoirs for other Flaviviruses.

Virus	Tissue/Secretion	Duration
Siberian Tick Borne encephalitis (TBEV)	Brain/Nervous system	10 years ^44^
Japanese Encephalitis Virus (JEV)	CSF	>3 weeks post- infection ^45^
	PBMCs	8–9 months after infection ^46^
West Nile Virus (WNV)	Blood/organ transplants	>40 days ^47– 50^
	Urine	6.5 years ^51^

Another potential concern of ZIKV tissue reservoirs (with low levels of ZIKV) is triggering of sub-neutralizing antibodies leading to antibody-dependent enhancement (ADE) of subsequent Flavivirus infections and potential interplay with other mosquito born Flaviviruses. Recently, ADE of ZIKV by sub-neutralizing Dengue virus antibodies has been reported ^36, 37^. Considering the similar target organ affinity (e.g. nervous tissue) observed for other Flaviviruses (e.g. West Nile Virus, Dengue Virus) ^33^, such a concern cannot be ruled out.

## Challenges for the development of countermeasures against ZIKV

The presence of tissue reservoirs and detection of the virus for varying periods of duration, after initial infection, in the absence of the apparent symptoms, poses new challenges for screening of populations. In 2013 during the French Polynesian Zika outbreak, differential virus positivity was observed between the serum, saliva and urine samples collected from 182 patients. Saliva but not serum, from 35 patients was Zika positive. In contrast, serum but not saliva from 16 other patients was Zika positive ^37^. Considering such variability in different biological samples from patients, paired serum and urine samples are considered to be of primary diagnostic importance (CDC guidance for US laboratories testing for Zika virus infections).

Interestingly, in animal studies, low levels of Zika were observed in the body secretions compared to the serum ^38^. Such low levels of ZIKV in the body secretions near the detection limits and invasive processes needed to collect samples (eg. CSF, nervous tissue), suggest currently available detection assays may or may not be suitable to identify persistent ZIKV infections and quantify the tissue reservoirs. As per the CDC information, there are currently no FDA-authorized assays for Zika virus testing of tissue specimens, including fetal and placental tissue, suggesting the need for additional efforts towards such diagnostic assays.

FDA has issued Emergency Use authorization of several diagnostic tools for Zika virus. These include both antibody based and nucleic acid testing (NAT) kits, which are currently distributed to qualified laboratories by the CDC ( Table 3).

**Table 3.** Zika virus diagnostic test kits available through Emergency Use Authorization of the FDA.

Kit	Detection method	Vendor
DPP Zika IgM Assay System	Antibody	Chembio Diagnostic Systems, Inc.
ADVIA Centaur Zika test	Antibody	Siemens Healthcare Diagnostics Inc
CII-ArboViroPlex rRT-PCR Assay	NAT	Columbia University
TaqPath Zika Virus Kit	NAT	Thermo Fisher Scientific
LIAISON ^®^ XL Zika Capture IgM Assay	Antibody	DiaSorin Incorporated
Gene-RADAR ^®^ Zika Virus Test	NAT	Nanobiosym Diagnostics, Inc
Zika ELITe MGB ^®^ Kit U.S.	NAT	ELITechGroup Inc. Molecular Diagnostics
Abbott RealTime Zika	NAT	Abbott Molecular Inc.
Zika Virus Detection by RT-PCR Test	NAT	ARUP Laboratories
Sentosa ^®^ SA ZIKV RT-PCR Test	NAT	Vela Diagnostics USA, Inc.
ZIKV Detect™ IgM Capture ELISA	Antibody	InBios International, Inc
xMAP ^®^ MultiFLEX™ Zika RNA Assay	NAT	Luminex Corporation
VERSANT ^®^ Zika RNA 1.0 Assay (kPCR) Kit	NAT	Siemens Healthcare Diagnostics Inc
Zika Virus Real-time RT-PCR Test	NAT	Viracor Eurofins
Aptima ^®^ Zika Virus Assay	NAT	Hologic, Inc
RealStar ^®^ Zika Virus RT-PCR Kit U.S.	NAT	Altona Diagnostics
Zika Virus RNA Qualitative Real-Time RT-PCR	NAT	Quest Diagnostics Infectious Disease, Inc
Zika MAC-ELISA	Antibody	CDC
Trioplex Real-time RT-PCR Assay	NAT	CDC

NAT: Nucleic Acid based detection Test

Antibody based ZIKV detection methods are suggestive of past or recent exposure to Zika and may not necessarily indicate the presence of the virus in the body. In addition, antibody based methods are suitable only for serum, whereas NAT might be suitable for all types of samples including serum and bodily fluids. Due to the direct detection of the virus, NAT-based detection kits might be well suited for the identification of persistent infections. A rapid and high-throughput method for detecting ZIKV RNA using transcription-mediated amplification technology, based Aptima Zika virus assay (Hologic, Marlborough, MA) is described to detect 11.5 to 17.5 genome copy equivalents in serum and urine ^39^. A recent report suggests the utility of isothermal amplification based point-of-care diagnostic technology for rapid detection of ZIKV in the saliva ^40^. The performance of such kits in the clinic remains to be seen. Additional research might shed some light on the acceptable standard for such an effort.

ZIKV tissue reservoirs bring in additional challenges for drug or vaccine development. According to the WHO vaccine pipeline tracker, there are many ZIKV vaccine candidates currently being tested. These include four DNA vaccines (one from GeneOne Life Sciences and three from NIAID), four inactivated whole virus vaccines (3 from NIAID and 1 from BIDMC), one peptide (NIH), one mRNA (Moderna), and one recombinant MV-Zika-101 (Themis Bioscience). Two of these candidates- VRC-ZKADNA090-00-VP (NIAID) and mRNA 1325 (Moderna) are currently recruiting patients for phase 2 trials.

To be effective, potential treatments for ZIKV might have to achieve appropriate tissue concentrations in these reservoirs. This might be limited by sufficient bioavailability of therapeutics in the tissue reservoirs and penetration of the blood brain barrier (BBB). Similar concerns for placental barrier and challenging anatomical locations (e.g. the eyes, kidney, and testes) create additional hurdles for elimination of viral reservoirs. Prophylactic vaccine strategies should be capable of not only preventing infection, but also prevent the establishment of viral reservoirs. In addition, potential vaccine candidates should be capable of inducing a potent neutralizing antibody response to mitigate the possibility of ADE mentioned above.

## Concluding remarks

ZIKV animal models (mice, monkeys) could serve as valuable tools in evaluating the efficacy of therapeutic and prophylactic strategies in eliminating ZIKV tissue reservoirs and prevent shedding of the virus. Additional questions such as relative difference between ZIKV strains to persist in the tissue reservoirs, relative size of different tissue reservoirs (brain, eyes, testes), role of host factors in persistence (tissue reservoir formation), kinetics of virus persistence, and phenotypic and genotypic characterization of the virus in tissue reservoirs, will require additional studies. Formation of ZIKV task force by the Global Virus Networks ^41^ is a welcome move that might be helpful in addressing some of these issues. There is a need for a publically available database (suitable for meta-analysis) to compare long-term kinetics of ZIKV infections and persistence in both animal models and clinical settings. Together with additional research, this might reveal the impact of ZIKV tissue reservoirs on future outbreaks, and drug and vaccine development.
