# Supplementary material for: Zika virus reservoirs: Implications for transmission, future outbreaks, drug and vaccine development
Source: F1000Res. 2017 Oct 17;6:1850. [Version 1] doi: 10.12688/f1000research.12695.1 (PMC5710464; doi:10.12688/f1000research.12695.1)
Supplement: Peer review history

# Peer review: Referee response for version 1

**DOI:** 10.5256/f1000research.13748.r28041

**Published:** 2017-11-27

**Authors:** Michael P Busch, Mars Stone

**Affiliations:** 1 Blood Systems Research Institute, San Francisco, California, USA; 2 University of California, San Francisco, California, USA

Kalkeri and Murthy offer a valuable brief review of tissue reservoirs and its implications for transmission and treatment of Zika infection.  Since studies of persistence of Zika virus infection in tissue reservoirs are limited in humans and hence not well understood, insights from rhesus macaque and mouse models of Zika infection are important to characterize persistence and its relevance to transmission and pathogenesis, as well as addressing challenges in treatment and diagnosis.

 Not mentioned in the review is the finding of persistence in whole blood, and in particular RBCs, after clearance from plasma (several publications) and the potential risk for transmission through bone marrow, cord blood and peripheral blood stem cell transplants.  Transfusion transmission has been documented as another mode of transmission.

 Co-circulation of related Flaviviruses in Zika endemic areas is a concern for ADE of subsequent antigenically similar viruses as mentioned in the review. However evidence for ADE in humans is lacking.  ADE from previous Dengue virus exposure has been demonstrated in vitro and in mouse models, but not in rhesus macaques and is not confirmed by human epidemiological studies based on several recent publications.  Conclusions about ADE should therefore be made with caution.

 Briefly mentioned are issues with serological diagnostics, which are challenging due to cross reactivity and anamnestic response in serial infections by similar viruses as well as Arboviral vaccination. There are limitations with many existing assays, however newer assays have been more successful in differentiating Zika from Dengue.  Access to samples from ZIKV/DENV infected macaques could allow for advances in this important area.

 Reference 37 (ZIKV in saliva) is incorrectly placed in the context of ADE second paragraph of page 3.

 Suggest adding references at the bottom.

We have read this submission. We believe that we have an appropriate level of expertise to confirm that it is of an acceptable scientific standard.

## References

[1] MussoDRichardVTeissierAStoneMLanteriMCLatoniGAlsinaJReikRBuschMPRecipient Epidemiology and Donor Evaluation Study REDS-III ZIKV Study Group: Detection of Zika virus RNA in semen of asymptomatic blood donors. *Clin Microbiol Infect*.2017; 10.1016/j.cmi.2017.07.006 10.1016/j.cmi.2017.07.006 28711704PMC6016846

[2] MussoDRocheCRobinENhanTTeissierACao-LormeauVM: Potential sexual transmission of Zika virus. *Emerg Infect Dis*.2015;21(2) : 10.3201/eid2102.141363 359-61 10.3201/eid2102.141363 25625872PMC4313657

[3] MussoDRocheCNhanTXRobinETeissierACao-LormeauVM: Detection of Zika virus in saliva. *J Clin Virol*.2015;68: 10.1016/j.jcv.2015.04.021 53-5 10.1016/j.jcv.2015.04.021 26071336

[4] BestKGuedjJMadelainVde LamballerieXLimSYOsunaCEWhitneyJBPerelsonAS: Zika plasma viral dynamics in nonhuman primates provides insights into early infection and antiviral strategies. *Proc Natl Acad Sci U S A*.2017;114(33) : 10.1073/pnas.1704011114 8847-8852 10.1073/pnas.1704011114 28765371PMC5565429

[5] AidMAbbinkPLaroccaRABoydMNityanandamRNanayakkaraOMartinotAJMoseleyETBlassEBorducchiENChandrashekarABrinkmanALMolloyKJettonDTartagliaLJLiuJBestKPerelsonASDe La BarreraRALewisMGBarouchDH: Zika Virus Persistence in the Central Nervous System and Lymph Nodes of Rhesus Monkeys. *Cell*.2017;169(4) : 10.1016/j.cell.2017.04.008 610-620.e14 10.1016/j.cell.2017.04.008 28457610PMC5426912

[6] MachadoCPereiraBFelixAOliveiraMDarrigoLde SouzaMPatonENevesFColturatoVSimoesB: Zika and chikungunya virus infections in hematopoietic stem cell transplant recipients and oncohematological patients. *Blood Advances*.2017;1(10) : 10.1182/bloodadvances.2016003285 624-627 10.1182/bloodadvances.2016003285 PMC572835129296705

[7] MansuyJMMengelleCPasquierCChapuy-RegaudSDelobelPMartin-BlondelGIzopetJ: Zika Virus Infection and Prolonged Viremia in Whole-Blood Specimens. *Emerg Infect Dis*.23(5) : 10.3201/eid2305.161631 863-865 10.3201/eid2305.161631 28257281PMC5403064

[8] MurrayKOGorchakovRCarlsonARBerryRLaiLNatrajanMGarciaMNCorreaAPatelSMAagaardKMulliganMJ: Prolonged Detection of Zika Virus in Vaginal Secretions and Whole Blood. *Emerg Infect Dis*.23(1) : 10.3201/eid2301.161394 99-101 10.3201/eid2301.161394 27748649PMC5176245

[9] McCrackenMKGromowskiGDFribergHLLinXAbbinkPDe La BarreraREcklesKHGarverLSBoydMJettonDBarouchDHWiseMCLewisBSCurrierJRModjarradKMilazzoMLiuMMullinsABPutnakJRMichaelNLJarmanRGThomasSJ: Impact of prior flavivirus immunity on Zika virus infection in rhesus macaques. *PLoS Pathog*.2017;13(8) : 10.1371/journal.ppat.1006487 e1006487 10.1371/journal.ppat.1006487 28771605PMC5542404

[10] TerzianASchanoskiAMotaMda SilvaREstofoleteCColomboTRahalPHanleyKVasilakisNKalilJNogueiraM: Viral Load and Cytokine Response Profile Does Not Support Antibody-Dependent Enhancement in Dengue-Primed Zika Virus–Infected Patients. *Clinical Infectious Diseases*.2017;65(8) : 10.1093/cid/cix558 1260-1265 10.1093/cid/cix558 PMC584910329017246

[11] CarrollTLoMLanteriMDutraJZarbockKSilveiraPRourkeTMaZMFrittsLO'ConnorSBuschMMillerCJ: Zika virus preferentially replicates in the female reproductive tract after vaginal inoculation of rhesus macaques. *PLoS Pathog*.2017;13(7) : 10.1371/journal.ppat.1006537 e1006537 10.1371/journal.ppat.1006537 28746373PMC5546709

# Peer review: Referee response for version 1

**DOI:** 10.5256/f1000research.13748.r27070

**Published:** 2017-10-30

**Authors:** Sunil K Joshi

**Affiliations:** 1 Cellular Immunology Lab, Frank Reidy Research Center of Bioelectrics, Old Dominion University, Norfolk, VA, USA

This report is very important in terms of ZIKA as reservoir. Authors have done excellent work in writing this unique review and will enhance our understanding of ZIKA transmission via reservoir infection.

 Recent reports indicating that detection of ZIKV in the bodily secretions of infected patients, such as saliva, urine, semen and vaginal secretions, breast milk and conjunctival fluid is an important public health concern, particularly reservoir ZIKV still can cause spread of the disease.

I have read this submission. I believe that I have an appropriate level of expertise to confirm that it is of an acceptable scientific standard.

## References

[1] MunjalAKhandiaRDhamaKSachanSKarthikKTiwariRMalikYSKumarDSinghRKIqbalHMNJoshiSK: Advances in Developing Therapies to Combat Zika Virus: Current Knowledge and Future Perspectives. *Front Microbiol*.2017;8: 10.3389/fmicb.2017.01469 1469 10.3389/fmicb.2017.01469 28824594PMC5541032
